# Evolution of specialist treatment of gaming disorder and internet addiction in Japan

**DOI:** 10.3389/fpsyt.2025.1577135

**Published:** 2025-12-10

**Authors:** Takashi Kitayuguchi, Takanobu Matsuzaki, Kotaro Nishimura, Satoko Mihara, Susumu Higuchi

**Affiliations:** Treatment of Internet Addiction and Research Department, National Hospital Organization Kurihama Medical and Addiction Center, Yokosuka, Japan

**Keywords:** gaming disorder, internet addiction, treatment facility, treatment program, treatment goal, autism spectrum disorder, attention deficit hyperactivity disorder, Japan

## Abstract

**Introduction:**

Despite a relatively short history in the field of disorders, treatment evidence for gaming disorder (GD) and internet addiction (IA) has been gathered. Notwithstanding, in many countries, an understanding of treatment service delivery is lacking. In this study, we have investigated how treatment services at specialist facilities have evolved in Japan, historically and up to the current time, focusing on service delivery and treatment challenges at these facilities.

**Methods:**

We firstly identified facilities providing specialist treatment for GD and IA using mailed questionnaire surveys, with the assistance of mental health and welfare centers, in 2016, 2018, 2020 and 2023. In order to elucidate how treatment was delivered, together with the related treatment difficulties, additional questionnaire surveys were conducted in 2020 and 2023 with specialist treatment facilities, as identified by the aforementioned surveys.

**Results:**

The number of facilities showed a 4.5-fold increase between 2016 and 2023. The service delivery was initiated, maintained, and led by facilities in response to treatment and consultation demand. Despite this increase in number, the geographical distribution has been uneven. With regard to treatment goals, the vast majority of facilities have focused on encouraging activities other than gaming rather than simply reducing gaming time. Notably, between 2020 and 2023, there was a tendency away from generalized and toward specialized treatment programs. Additionally, specialists faced a range of difficulties in treatment delivery, often related to the high comorbidity rate of neurodevelopmental disorders. However, during this period, specialists became increasingly adept at managing these issues.

**Discussion:**

The results suggested that the service delivery system for GD and IA has rapidly developed, driven by treatment facilities in response to growing treatment and consultation demand, which may be characteristic of, or even unique to Japan. The results also suggested that specialization of treatment programs and the skills of specialists in managing difficulties in the course of treatment may have advanced in a relatively brief period of time. Challenges to be addressed include a widening gap between the numbers of treatment seekers and the capacity of treatment providers, together with the skewed geographical distribution of facilities.

## 1 Introduction

The term, internet addiction (IA) has been used since the late 1990s, but a globally approved definition has yet to be established. Internet gaming disorder (IGD) was included in the Fifth Edition of the Diagnostic and Statistical Manual of Mental Disorders as a “disorder warranting further study” (1).

Subsequently, gaming disorder (GD) was officially recognized as a disorder in the Eleventh Revision of the International Classification of Diseases, in May 2019 (2). Although, the study of GD and IA treatment is in the early stages due to the relatively short history of these disorders, treatment-related evidence has been gathered.

According to a recent review and meta-analysis, the global prevalence of IGD has been estimated at 2.96%, and 1.96% when considering only studies with more stringent sampling criteria (3, 4). The prevalence among males is approximately 2.3 times higher than that of females (3, 4). With respect to gender differences, it has been suggested that, during the developmental trajectory of IGD, high levels of impulsivity, deficits in inhibitory control, and aggressive behaviors are more frequently observed among males than females. In contrast, loneliness and difficulties in emotional regulation appear to be more prevalent among females with IGD compared to their male counterparts (5).

With regard to screening and assessment scales of IGD, although several assessment tools such as the Seven-Item Game Addiction Scale (GAS-7), the Assessment of Internet and Computer Addiction Scale-Gaming (AICA-S Gaming), the Ten-Item Internet Gaming Disorder Test (IGDT-10), the Internet Gaming Disorder Scale (Lemmens IGD-9), and the Internet Gaming Disorder Scale-Short Form (IGDS9- SF) have shown acceptable psychometric properties, including internal consistency and test-retest reliability (6), there remains no consensus on a gold-standard instrument (7). Screening instruments developed in accordance with the ICD-11 diagnostic criteria include the Gaming Engagement Screener Test (GAMES test) (8) and the ICD-11-based Gaming Disorder Scale for Adolescents (GADIS-A) (9).

A range of factors have been identified as contributing to the development and maintenance of GD, including gender, impulsivity, depression, comorbid psychiatric conditions such as attention- deficit/hyperactivity disorder (ADHD), anxiety, stress, time spent gaming, escapism motives, low self- esteem, and feelings of loneliness (10–13). Regarding the relationship between GD and emotion regulation, it has been suggested that video games may serve as an avoidant strategy to suppress emotional expression, and that greater severity of GD may be associated with increased difficulties in emotion regulation (14). Furthermore, individuals who play video games and experience various psychological problems such as depression and anxiety are more likely to immerse themselves in gaming as a means of coping with daily challenges, alleviating stress, or improving their mood (15).

Ropovik et al. proposed that the motivations underlying gaming behavior may be influenced by specific emotional states. Loneliness may elicit social motivations, anger may elicit achievement-related motivations, and real-life stress may elicit escapism-related motivations (13).

Regarding protective factors of GD, peer relationships and healthy family functioning have been suggested as potential factors against excessive gaming. Although empirical evidence remains limited, a sense of safety and belonging at school has also been identified as a possible protective factor (12).

Like other addictions, treatment of GD and IA consists of psychosocial treatment and pharmacotherapy, with the former the main modality. Regarding psychosocial approaches, cognitive behavioral therapy (CBT) based treatment has been the most comprehensively studied approach and its efficacy has been confirmed (16–18). For example, Wölfling et al. conducted a randomized controlled trial (RCT) to examine the efficacy of 15 weekly group and up to 8 fortnightly individual sessions with IGD cases, using a short-term CBT program (19). The results showed that the remission rate based on a self-report assessment scale was significantly higher than that of the wait list control.

(19). Other promising psychosocial approaches include: mindfulness, relapse prevention, abstinence protocols and family therapy (16, 17, 20, 21). With regard to pharmacotherapy, no medicine has been approved for use for GD and IA by any national government. However, antidepressants such as bupropion and escitalopram have been suggested to be effective (22). In cases where comorbidities such as attention deficit hyperactivity disorder (ADHD) and depression are present, pharmaceutical intervention was reported to be an effective option (23). Some studies suggested that pharmacotherapy for ADHD improved symptoms not only of ADHD but also GD in comorbid cases (24). In addition to these treatment modalities, emerging approaches involving non-invasive brain stimulation have been studied. For example, electro-acupuncture, transcranial direct current stimulation and repetitive transcranial magnetic stimulation have been researched and suggested to be effective in reducing GD and IA symptoms, as well as modulating brain activity (16, 17, 25).

In contrast to the increased number of studies on the treatment approaches for GD and IA, far less is known about treatment service delivery systems and treatment programs conducted in clinical settings. While King et al. explored the treatment service delivery in four countries (26), the study focused on the main specialist treatment centers and challenges these centers faced, specifically during the COVID-19 pandemic. Long et al. examined public health approaches and policy changes after the inclusion of gaming disorder in ICD-11 in several countries including Australia, China, Italy, India, Switzerland, and USA (27). Specialist treatment services are emerging rather than fully formed and so, have received less attention. However, most countries have developed manpower in the treatment of GD (27).

In Japan, due to the recent nature of delivery of specialist treatment services for GD and IA, a formal list of medical facilities for these disorders had not been compiled until 2015. In order to address this situation, we have conducted a series of surveys to identify specialist treatment facilities. The subsequent list we complied has been published on our center’s website for individuals and families affected by GD and IA, to provide options for facilities. In addition, we conducted another set of surveys to investigate how treatment was conducted at these facilities. The results suggested that the approaches by Japanese providers have rapidly developed. Notably, these has been driven by the treatment facilities themselves in response to the growing treatment and consultation demand from affected individuals, their families, as well as from other facilities and the wider community.

## 2 Materials and methods

### 2.1 Surveys to identify specialist treatment facilities

A short questionnaire was mailed to all mental health and welfare centers in Japan (n=67) in order to compile a list of medical facilities providing specialist treatment for GD and IA in locations served by these centers. Mental health and welfare centers are organizations that support the welfare of individuals with mental health disorders, as stipulated by the Act on Mental Health and Welfare for the Mentally Disabled. At least one organization has been established by local government in each prefecture and government ordinance designated city. These organizations provide official information related to medical treatment services for mental health disorders, which are available in the catchment area. To the authors’ knowledge, this is the best way to identify a complete and unbiased list of facilities which most likely provide specialist treatment of GD and IA in Japan.

After receiving responses from all centers to our questionnaire, we developed a provisional list of specialist facilities for GD and IA treatment in Japan. We also conducted an additional survey, cognizant of the fact that we may have had an inaccurate or incomplete understanding of the IA and GD treatment options at some centers. A questionnaire focusing on the provision of specialist treatment services was sent to each treatment facility on the list. The final list was then completed based on the response from each facility. We have conducted two set of surveys in each of 2016, 2018, 2020 and 2023, and the same surveys will again be conducted in 2025. The list has been made available on our center’s website to provide patients and their families with information on treatment options at the local level.

### 2.2 Surveys on treatment at specialist facilities

In addition to identifying specialist treatment facilities for GD and IA, we conducted a survey on the treatment provided at these facilities. The subjects were 127 treatment facilities identified by the aforementioned survey in 2023. A questionnaire was mailed to these facilities, completed, and returned. The content of the questionnaire comprised 1) whether specialist medical treatment was provided, 2) reasons for initiating specialist treatment, 3) current status of patients, 4) psychiatric comorbidities 5) treatment programs including pharmacotherapy, 6) inpatient treatment, 7) difficulties in treating GD and IA, and 8) treatment target and social resources. The questionnaires used in these surveys were developed based on a series of discussions by experts in the field of behavioral addictions. The content of the questionnaire is believed to reflect the actual GD and IA treatment environment.

Eighty-one facilities responded to the survey; a response rate of 64%. In 2020, a survey was conducted that was similar in process and content to the 2023 survey. For the earlier survey, the number of subjects was 89 and 68 facilities responded (response rate: 76%). We were able to compare certain results in the 2020 and 2023 surveys.

### 2.3 Statistical analysis

The data were analyzed using SAS 9.4 for Windows (SAS Institute, Cary, NC, United States). Comparison of the rate of questions endorsed between the 2020 and 2023 surveys was tested using the chi-square test. The statistical significance was set at *P* < 0.05, and the tendency for significance was set at *P* < 0.1 (two-tailed).

## 3 Results

### 3.1 Trend in specialist treatment facilities

**Figure 1:**
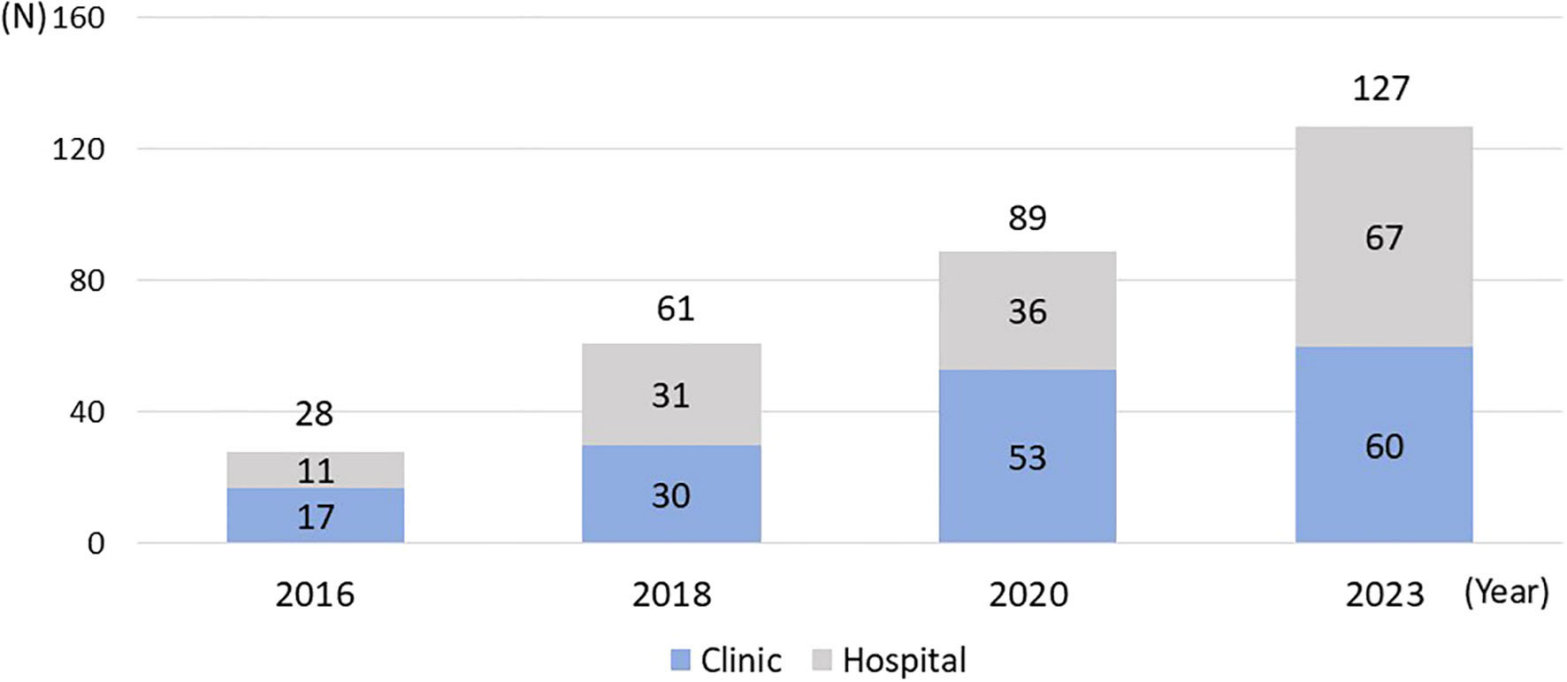
Change in the number of facilities providing specialist treatment of GD and IA in Japan.

**Figure 2:**
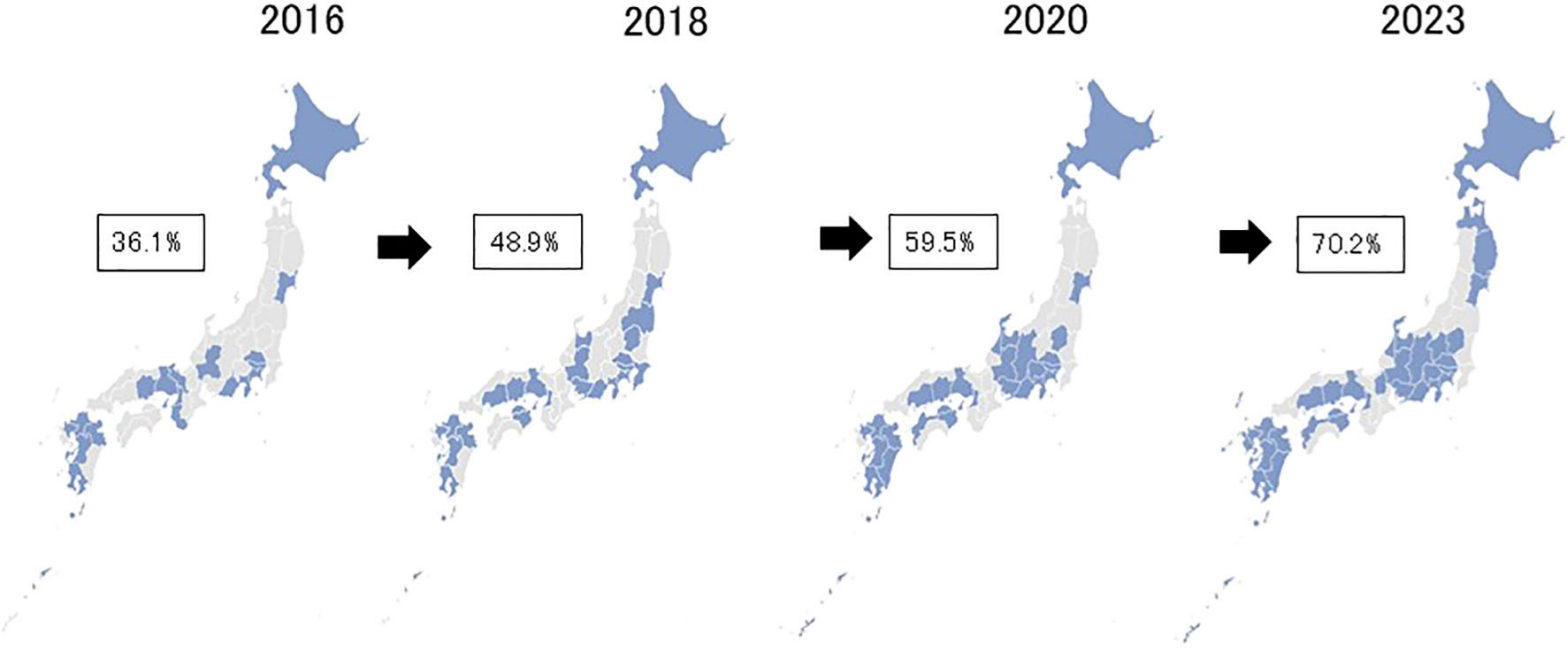
Advancement in geographical distributions of specialist treatment facilities for GD and IA from 2016 through 2023 in Japan. Footnote. The percentages in the figure represent those of prefectures having at least one specialist facility in respective years.

The number of clinics and hospitals providing specialist treatment for GD and IA has constantly increased since the first survey in 2016 (**Figure 1**). We observed a nearly 4.5 -fold increase in the total number of facilities between 2016 and 2023. **Figure 2** shows the geographical distribution of these facilities. In Japan, there are 47 prefectures, comparable to states or provinces in other countries. As the number of facilities has grown, it has become increasingly likely that a given prefecture will have at least one specialist treatment facility. In 2016, 36% had such a center, but by 2023 the percentagewas 70%. Characteristically, prefectures still lacking specialist facilities at that point were in rural and/or less densely-populated prefectures.

### 3.2 Surveys on treatment at specialist facilities

#### 3.2.1 Characteristics of treatment facilities and patients

In 2023, the reply to one of the multiple-choice questions revealed that 86% of specialist treatment was provided by a department of psychiatry, followed by departments of child and adolescent psychiatry (46%). About 5% of services were provided by departments of pediatrics. There was little difference regarding treatment by medical departments between 2020 and 2023.

Regarding the reasons why facilities began providing specialist treatment, the most frequent multiple-choice response was “increase in the number of consultations from families”, followed by “increase in the number of patients” (**Table 1**). Notably, many facilities began offering specialist treatment as a response to demand for treatment from patients, families, other facilities, or the community, rather than at the request of local or national government.

**Table 1:** Current status of specialist treatment facilities for GD and IA (n=81)^a^.

Reasons for initiating to provide specialist treatment^a,b^	n	(%)
Increase in number of patients with GD/IA	45	(55.6)
Increase in number of consultations from families of patients	52	(64.2)
with GD/IA Interest in treatment of GD/IA	31	(38.3)
Request from other medical and consultation facilities	16	(19.8)
Request from the community	14	(17.3)
Current trend in patients with GD/IA^a,b^	n	(%)
Increase in number of patients	47	(58.0)
Increase in number of young children (younger than 12 years old)	36	(44.4)
Increase in SNS-related problems	30	(37.0)
Increase in video-related problems	26	(32.1)
Increase in smartphone-related problems	36	(44.4)

^a^Data are from the 2023 survey.

^b^Multiple choice questions.

Regarding the trend in patients with GD/IA who visited the facilities, 58% responded that the number of outpatients had increased, and 44% had identified an increase in young children (12 years old or younger) (**Table 1**). About 30%-40% of facilities had observed an increase in the number of patients with smartphone, SNS, or video-related problems.

We also enquired about the comorbidity of patients with GD/IA: “In cases with GD/IA your facilities have treated, what are the most frequent diagnoses of comorbidity? In order of frequency, please answer three, a) most common, b) second most common and c) third most common”. The results showed that a number of facilities endorsed autism spectrum disorder (ASD) and attention deficit hyperactivity disorder (ADHD) as the most and second-most common comorbid psychiatric disorder (**Table 2**). The rates were far higher than for other comorbidities, including: depression, bipolar disorder, anxiety disorder and obsessive-compulsive disorder. These results strongly suggest that comorbidity rates of ASD and ADHD were quite high in clinical GD and IA cases.

**Table 2:** Psychiatric comorbidity of GD and IA^a,b,c^.

Frequency	ASD	ADHD	Depression	BPD	Anxiety	OCD	Others
First	42	27	1	1	0	1	7
Second	22	40	4	3	4	2	1
Third	9	5	22	4	19	3	6

^a^Data are from the 2023 survey.

^b^Question asked: In cases with GD /IA your facilities have treated, what are the most frequent diagnoses of comorbidity? In order of frequency, please answer three (1). Most common, (2) Second most common, (3) Third most common.

^c^ASD (autism spectrum disorder, ADHD (attention deficit hyper activity disorder), BPD (bipolar disorder), OCD (obsessive compulsive disorder.

#### 3.2.2 Treatment provided at specialist facilities

**Figure 3:**
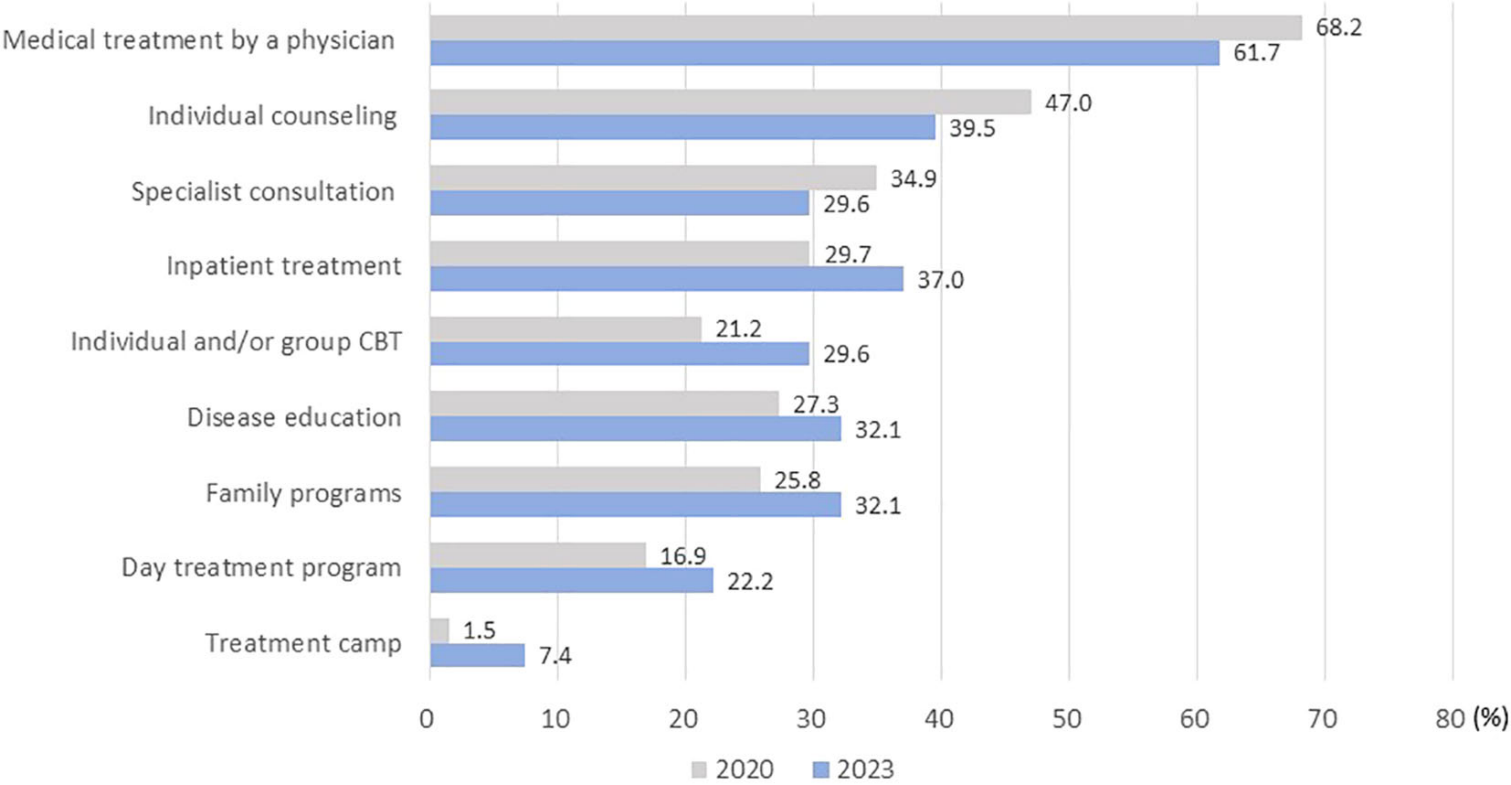
Change in treatment programs for GD and IA provided at specialist treatment facilities between 2020 and 2023.Footnote. None of the comparisons in the figure reached a statistically significant level.

**Figure 3** illustrates changes in treatment programs between 2020 and 2023. In 2020, generalized outpatient care, such as medical treatment by a physician and counseling was most frequently provided. However, between 2020 and 2023, there was a shift away from generalized programs and toward treatment specifically targeting GD and IA. The latter included individual and group cognitive behavioral therapy (CBT), disease education, family programs, and treatment camps. Facilities tended to provide more specialized programs in 2023 compared to 2020.

Regarding the treatment target for GD, nearly all facilities (94%) replied that a reduction in gaming was the objective, rather than completely refraining from playing games (**Table 3**). Interestingly, the vast majority of facilities (83%) prioritized “improvement and enrichment of activities other than gaming” in the treatment of GD. In contrast, only a small number of facilities thought that “reduction in gaming time” and “improvement in GD symptoms” were most important.

**Table 3:** Treatment target of patients with GD set by specialist facilities (n=75)^a,b^.

Treatment target in gaming	n	(%)
Reduction	72	(96.0)
To refrain from playing	3	(4.0)
Most important aspect in treatment of GD	n	(%)
Reduction in gaming time	7	(9.3)
Improvement in GD symptoms	5	(6.7)
Improvement and enrichment of activities other than gaming	62	(82.7)
Others	1	(1.3)

^a^Data are from the 2023 survey.

^b^6 facilities did not respond to these questions.

**Figure 4:**
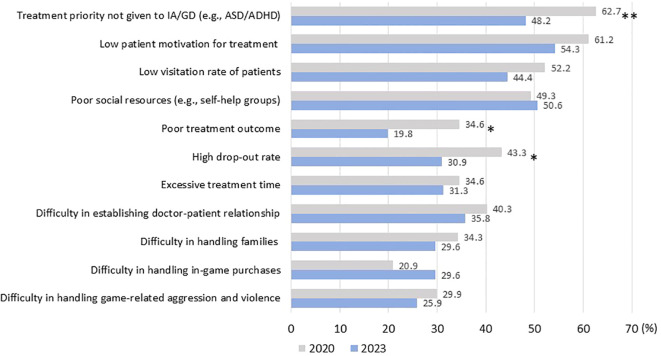
Change in difficulties of IA and GD treatment experienced by specialists between 2020 and 2023 Footnote. *P < .10, **P < 0.05 (by chi-square test) ASD: autism spectrum disorder. ADHD: attention deficit hyperactivity disorder.

**Figure 4** shows the difficulties specialists faced in treating GD and IA. In 2020, the highest percentage was reported as “treatment priority was different from GD or IA”. This means that prioritized treatment was given to comorbid psychopathologies such ASD and ADHD, reflecting theextremely high comorbidity rates of these disorders. Other reported difficulties included low patient motivation for treatment, low patient visitation rates, and poor social resources. Notably, for the majority of items related to treatment provided by facilities, the rate of difficulty tended to decrease between 2020 and 2023.

In 2023, inpatient treatment was available at 30 (37%) facilities. Pharmacotherapy was utilized for comorbid psychopathologies at 63 (78%) facilities and the off-label use of medicines was used for the treatment of GD/ IA at 7 (9%) facilities, although details of medication were not investigated in the survey. About two-thirds of facilities (63%) complained of an absence of self-help groups for GD/IA. Instead, 10 (12%) facilities used self-help groups for other addictions, such as Gamblers Anonymous, for patients with GD/IA.

## 4 Discussion

Because GD and IA are relatively new diseases, relatively little information has been gathered on the treatment of these diseases in real clinical settings. In this study, we have reported on how treatments for GD and IA have been developed and conducted at specialist facilities in Japan.

The results of our study show that the number of medical facilities providing specialist treatment has significantly increased since 2016. However, the treatment facilities listed in this study are most likely part of specialist facilities for the treatment of GD and IA. This study identified facilities treating GD and IA with the assistance of the mental health and welfare centers in each prefecture and large city. However, according to a recent survey conducted with child and adolescent psychiatrists, certified by the Japanese Society for Child and Adolescent Psychiatry, many young people with problems related to excessive use of gaming and the internet also visited their facilities for treatment for GD and IA (28). The overwhelming majority of these facilities were not part of our data gathering efforts. Similarly, pediatricians often treated subjects affected by GD and IA (29). Our findings strongly suggest that the demand for treatment has outpaced treatment capacity, notwithstanding the significant expansion of services.

One of the unique features of the rapid increase in specialist treatment capacity in Japan are the reasons for the emergence of treatment at these facilities. The main reasons were an increase in the treatment and consultation demand from patients, their families, and other medical and consultation facilities. In other words, the increase has been led by individual treatment providers, rather than by government. Our center has shared treatment expertise on GD and IA and offered consultation via training seminars since 2014. Nearly 1,600 experts have undergone training as of the end of 2023. This capability development may have contributed to the voluntary initiation of specialist treatment at these facilities.

Despite the expansion of specialist facilities across prefectures, the geographical distribution has been uneven. Facilities are relatively concentrated in urban prefectures, and rural and less populated prefectures have been left behind. National and local governments may have to take the lead in addressing the absence of treatment services for those in need.

Recovery from GD as a concept is not explicitly mentioned in the existing literature (30). Rather, changes in subjects’ disorders are described in terms of decreases/reductions in symptom severity, or improvement/increases. However, when it comes to the treatment of cases in clinical settings, the treatment goal is usually either a reduction in gaming or else to play games in an appropriate manner, and almost all facilities treated patients based on this goal (31). In addition, the vast majority of facilities also sought to improve and enrich activities other than gaming as a mean of reducing gaming. Gaming behavior and related problems are usually quite unstable if a reduction of gaming is set as a treatment goal during the course of treatment. Instead, one of the most effective strategies to achieve stable improvement is to replace gaming time with real world activities, such as school, sports, or employment. If gaming falls to the second-highest priority or lower in a patient’s life, stable improvement can usually be achieved and consolidated.

Comparison of treatment programs between 2020 and 2023 revealed that the rates of generalized programs provided at facilities tended to decrease. On the contrary, the rates of more specialized programs targeting GD and IA tended to increase. Historically, group CBT, disease education, family programs and specialized day treatment programs have been conducted as specialized treatment programs for substance dependence in Japan, and it was believed these could be incorporated in the treatment programs for GD and IA. A treatment camp is a unique program for the improvement of GD and IA. Our center began to offer this service to GD and IA outpatients in 2014. The number of treatment facilities offering this program has gradually increased, due to its effectiveness in treating GD and IA, and camps have been positively received by participants (32).

The experience of specialists in terms of treatment challenges generally improved between 2020 and 2023. This may mean that specialists became more adept at managing issues. These results suggest that specialized programs have been enriched and specialists better able to manage cases in a relatively brief interval, implying specialization at these facilities has enabled rapid progress.

ADHD and ASD have been reported as risk factors for GA and IA (33, 34). The high prevalence of both ASD and ADHD was reported in subjects with IA among both the general population and clinical samples in Japan (35, 36). From the perspective of treatment, the literature has reported that comorbidity of ADHD and ASD is associated with more severe symptoms and poorer treatment outcome in cases with GD and IA (33, 37, 38). In this study, treatment facilities reported that ADHD and ASD were highly prevalent among cases with GD and IA, although the real comorbidity rates were not investigated. This situation caused difficulties when specialists saw cases with GD and IA, resulting in poorer treatment outcome, which was reflected in the responses from facilities. Pharmacotherapy as a treatment of GD is underdeveloped and the Japanese government has yet to approve any specific medications to treat the disorder. Notwithstanding, a small number of facilities have utilized off-label medicines in GD treatment. In addition, pharmacotherapy of comorbid psychopathologies has been conducted at nearly 80% of facilities, in the wider context of high comorbidity rates of disorders, such as ASD and ADHD. Up-to-date guidelines to improve treatment among cases with psychopathologies, especially ADHD and ASD, need to be developed and disseminated.

Finally, the methodological limitations of this study have been discussed. Although we tried to identify specialist treatment facilities for GD and IA as completely and accurately as possible, those in pediatric and child and adolescent psychiatric fields may have been insufficiently covered. Secondly, this study focused on medical facilities. However, there are facilities outside the medical field, which are also providing specialist therapy. Thirdly, the clinical characteristics and treatment outcomes of individual cases at facilities were not investigated. We plan to conduct further surveys that include a broader range of facilities in other fields and to obtain relevant information from individual cases.

## 5 Conclusion

In this study, we conducted exercises on four occasions to identify treatment facilities proving specialist treatment for GD and IA in Japan, between 2016 and 2023. In addition, information on how treatment was delivered and the related difficulties faced by treatment specialists was also collected from these facilities.

The results revealed that firstly, the number of facilities rapidly and steadily increased during these seven years. Development and delivery of services was driven by facilities in response to treatment and consultation demand from affected individuals, their families, other treatment facilities, and the wider community. However, while the number of facilities has grown, the geographical distribution has been markedly uneven. Thirdly, with regard to treatment goals, the vast majority of facilities prioritized improvement of alternative activities rather than simply reducing gaming time. In addition, between 2020 and 2023, there was a shift from generalized to specialized treatment programs. Finally, specialists have faced a range of challenges in delivering treatment, often related to high comorbidity rate of ADHD and ASD. Encouragingly however, specialists have also become better able to manage these issues.

The results suggest that the service delivery system for GD and IA, driven by treatment facilities themselves has rapidly developed, which may be unique to Japan. The results also suggest that specialization of treatment programs and the skills of specialists to handle challenges in treatment may have advanced in a relatively short period of time. Notwithstanding this progress, issues that still need to be addressed include the escalating number of treatment seekers over the capacity of treatment providers, together with the skewed geographical distribution of facilities.

## Data Availability

The raw data supporting the conclusions of this article will be made available by the authors, without undue reservation.
